# Experimental Study of Multi-Angle Effects of Micron-Silica Fume on Micro-Pore Structure and Macroscopic Mechanical Properties of Rock-like Material Based on NMR and SEM

**DOI:** 10.3390/ma15093388

**Published:** 2022-05-09

**Authors:** Guanglin Tian, Hongwei Deng, Yigai Xiao, Songtao Yu

**Affiliations:** 1 School of Resources and Safety Engineering, Central South University, Changsha 410083, China; tgl15352006270@163.com; 2 Sinosteel Maanshan General Institute of Mining Research Co., Ltd., Maanshan 243000, China; 3 School of Emergency Management, Jiangxi University of Science and Technology, Ganzhou 341000, China; yusongtao92@163.com

**Keywords:** rock-like material, silica fume, porosity, fractal dimension, mechanical property

## Abstract

The experiment of rock-like material plays an important role in the simulation of engineering fractured rock mass. To further understand the influence of raw materials on rock-like materials, this paper carried out the indoor mechanical properties test and the micro-pore structure detection combining NMR and SEM. The effects of micron-silica fume (SF) on microporous structure parameters and macroscopic mechanical properties under different conditions of water–cement ratio (WCR) and sand–cement ratio (SCR) were discussed. The intrinsic relationship between parameters of different scales was analyzed. The experimental results showed that the porosity parameters of different radii gradually decreased with the increase in SF. The reduction rate of macroporous porosity was the greatest, and the decreasing rate of microporous porosity was the smallest. With the increase in SF, the microscopic characteristics of the internal surface changed from more pores, complex morphological distribution, rough surface to fewer pores, regular morphological distribution and flat and uniform surface. The box fractal dimension also showed a decreasing trend. Micro-pore structure makes a valuable contribution to the influence of SF on mechanical properties. The compressive strength and tensile strength increased with the increase in SF. The box fractal dimension and porosity of different radii were negatively correlated with mechanical strength. Different porosity parameters conformed to a good exponential relationship with mechanical properties. The research results can provide reference value and research space for subsequent rock-like material research.

## 1. Introduction

The actual geotechnical engineering is in a complex and changeable rock environment. Different types of rock masses are used as basic bearing units, and their structural strength and damage failure mechanism will directly affect the safety and stability of the engineering. After a long period of complex geological processes, there are unevenly distributed pores, micro-cracks and surface cracks inside and outside the natural rock body. In production activities, these micro-cracks and surface macroscopic cracks have important impacts on the safety and stability of geotechnical engineering. Thus, the relevant research on fractured rock mass has always been a hot spot for many experts and scholars at home and abroad. However, if the indoor mechanical test uses an original rock for fracture prefabrication, there are some defects such as high economic cost, the high difficulty of the operation and a long test cycle. Rock-like material is a composite hardening material in which a cementitious material undergoes a complex chemical reaction with an aqueous solution to form a composite hardening material that the reaction products wrap the aggregate. It can not only simulate the mechanical properties of the original rock by adjusting the experimental ratio but can also simulate the fractured rock mass of real engineering by prefabricated fissures with low difficulty in sample preparation, a wide range of raw material sources, low cost and safety. Research methods have been favored by more and more experts and scholars. Rock-like materials are composites formed by mixing different kinds of raw materials according to a certain proportion. Admixture is one of the important raw materials. It has a certain influence on the micro-pore structure and macro-mechanical properties. Therefore, to improve the understanding of rock-like materials, it is necessary to study the influence of admixture on their micro-pore structure and mechanical properties to provide a reference for further research.

Based on current studies of different scale parameters of cementitious materials by admixtures [1,2,3,4], silica fume (SF) is the highest frequency of admixture selection and has a very obvious effect on the design and development of concrete [5]. In recent years, the effects of SF on the pore structure and mechanical properties of porous materials such as concrete and fillers have also been a hot topic for many scholars. Karthik [6] and Liu [7] studied the influence of SF on the durability of concrete mechanical properties. The results showed that the mechanical properties and durability values increased with the increase in SF. Lu [8] carried out a study on the influence of SF and water–cement ratio (WCR) on the freeze-thaw resistance of ultra-high-performance concrete. The results found that SF can improve the freeze-thaw resistance at appropriate content, but the effect of WCR on the freeze-thaw resistance was stronger than that of SF. Elrahman [9] took ultra-light foamed concrete as the research object, obtaining the internal microscopic characteristics by Scanning Electron Microscope (SEM) and Computerized Tomography (CT) detection techniques. The influence of nano-silica fume on mechanical properties, shrinkage and adsorption was analyzed. The study showed that nano-silica fume could compact solid structures and reduce the contact area between bubbles, improving the compressive strength. The porosity, shrinkage and adsorption rate decreased with the increase in SF. In the influence of SF on hydration behavior and mechanical properties of portland cement of high sulfate resistance [10,11], SF with high pozzolanic activity directly participated in hydration reaction and improved the compressive strength of early materials. Moreover, the calcium hydroxide produced by hydration of portland cement stimulated the activity of SF and finally promoted the occurrence of secondary hydration reaction, improving the mechanical strength of concrete. In addition to its own pozzolanic reaction, SF also filled the useful space inside the material, improving the densification and homogenization of the concrete microstructure. Therefore its compressive strength increased, and the permeability decreased. Meanwhile, there was an obvious correlation between compressive strength and porosity [12,13]. Shi [14] studied the influence of SF on compressive strength, porosity and calcium hydroxide of ultra-high performance concrete. The results showed that the SF improved the compressive strength but also decreased the porosity and the content of calcium hydroxide. Furthermore, there was a negative correlation between porosity and compression strength, and the fitting relationship presented an exponential function.

According to the above research content, SF had corresponding influences on the micro-pore structure and macroscopic mechanical properties of cement-based materials. The above studies proved that there is an internal relationship between pore parameters and mechanical strength. Currently, the pore structure detection techniques of porous materials mainly include nuclear magnetic resonance (NMR), SEM, mercury intrusion experiment (MIP), CT, etc. Many researchers also used different detection methods mentioned above to measure pore parameters. Deng [15,16,17,18,19], Zhang [20] and Li [21,22,23,24] analyzed the pore evolution of different rock specimens and filling samples based on NMR. The correlation model between porosity and mechanical properties was proposed. Bu [25] used MIP to test the pore volume and pore size distribution of concrete under different proportions, establishing statistical models of mechanical strength, porosity and pore size distribution. Zarnaghi [26] used MIP to detect the microscopic pore structure of self-compactable concrete, calculating the fractal dimension of pore quality. The relationship between the fractal dimension and compressive strength was verified. The result showed that there is a good correlation between the compressive strength and fractal dimension. Hu [27] took SEM to detect the microscopic pore structure of backfill materials, discussing the influence of admixtures on pore structure and mechanical strength based on fractal theory. Qing [12] analyzed the internal correlation between fractal dimension and compressive strength and permeability of cement concrete by means of MIP and SEM. Zhang [28] conducted MIP on compacted concrete, respectively, discussing the fractal characteristics and morphology distribution characteristics of micro-pores. Hazra [29] took the local shale in India as the main research object and systematically discussed the internal correlation between fractal dimension, pore structure and thermal maturity.

Based on the above studies on the influence of SF on the micro-pore structure and mechanical properties, it can be seen that many experiments only analyze the influence of SF on parameters of different scales under specific conditions. The research did not discuss whether the SF has the same influence on parameters of different scales under different conditions. Additionally, the correlation analysis between micro-pore structures and macroscopic mechanical properties is also lacking. In addition, most experiments used a single detection method to test pore parameters rather than a combination of multiple methods. In view of this, in this paper, NMR and SEM were used to detect the microscopic pore parameters under different incorporation conditions of SF. The box fractal dimension was calculated from the electron microscope pictures of the specimen section, and the mechanical strength was tested by an indoor mechanical test. The influence of SF on pore parameters with different radii, fractal dimensions and mechanical properties under different conditions of WCR and sand–cement ratio (SCR) was analyzed. The internal relationship between mechanical properties and fractal dimension and porosity parameters of the different radii was discussed. The experimental results can provide a reference for subsequent rock-like experimental research.

## 2. Material and Experiment

### 2.1. Material and Sample Preparation

Based on current research results of rock-like material [1,2,3,4], ordinary portland cement (P.O42.5) from a wide source range was selected as the cementing material. The detailed composition of cement is shown in Table 1. The fine aggregate is quartz sand with a particle size of 0.5–1 mm and a density of 1.49 g/cm^3^. The material form is a yellow and white ball shape with a smooth texture. It can be fully wrapped by hydration products of the cementitious material. The admixture is SF and a naphthalene water-reducing agent. The added value of the naphthalene water-reducing agent is a constant value, and the shape is yellowish-brown powder. The specific parameters are shown in Table 2. The test solution is tap water. The SF selected in the test is micron-silica fume in the form of white powder. Its particle size is 1 μm, and its density range is 2.2–2.6 g/cm^3^. The content of SiO_2_ is about 99.1%. Detailed parameters are shown in Table 3.

In this test, the amount of SF powder is taken as the dependent variable, and the parameter variation range is 6%, 9% and 12% (the mass percentage of SF to cement). The experiments conditions of WCR and SCR are designed into 3 groups: WCR = 0.30 and SCR = 1.0, WCR = 0.33 and SCR = 0.7, WCR = 0.35 and SCR = 1.3, studying the influence of SF on microscopic pore characteristics and mechanical properties under different proportions of WCR and SCR. The rock samples in each plan include 2 batches (3 samples in each batch), which were used to obtain the compressive strength (diameter: 50 mm, height: 100 mm) and tensile strength (diameter: 50 mm, height: 50 mm). The experimental scheme is shown in Table 4.

During sample preparation, firstly, cement, quartz sand, pure silica powder, naphthalene reducer and aqueous solution were weighed proportionally. The different raw materials were thoroughly mixed in accordance with the test protocol that was designed. Secondly, the raw materials that were stirred were loaded into a prepared cylindrical mold and placed on a standard vibrating table in the laboratory for vibration until the slurry appeared on the surface. Finally, all specimens that had completed the vibration were left indoors for 2 days and nights. Then these were numbered sequentially after demolding and placed in an indoor standard maintenance box with a temperature of 22 °C and relative humidity of 98% for 28 days.

### 2.2. Experimental Programme

#### 2.2.1. Mechanical Property Testing

The mechanical properties test consists of two parts: the uniaxial compressive strength test and the tensile strength test. The compressive strength test equipment is a WHY−300/10 microcomputer-controlled automatic pressure testing machine with a measuring range of 300 kN, which is produced by Shanghai Hualong Test Instrument in China. In accordance with the Standard Test Specification [30] of the Water Resources and Hydroelectric Engineering Rock Test Code in China, the control mode is force control with a loading rate of 1 kN/s. The calculation formula is as follows:

(1) $$f_{cc}=\frac{F}{A}$$

In Equation (1), $f_{cc}$ is the compressive strength (MPa). F is the destructive load (N). A is the pressure-bearing area of the specimen (mm^2^). The test control method selected for the tensile strength test is also force control, and the loading rate is also 1 kN/s. The calculation formula is shown in Equation (2).

(2) $$\sigma_{t}=\frac{2P}{\pi DH}$$

In Equation (2), $\sigma_{t}$ is the tensile strength (MPa). P is the destructive load (N). D is the diameter of the specimen (mm), and H is the height of the specimen (mm).

#### 2.2.2. Micro-Pore Structure Testing

**Figure 1:**
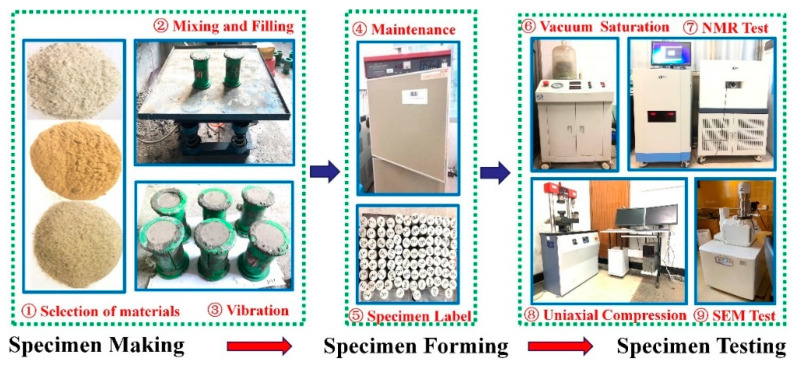
Experimental Process.

The microporous structure test is divided into two parts: porosity test (NMR) and surface micro-characteristic test (SEM). The test instrument selected for porosity is the MesoMR23-060 H rock NMR analysis system produced by Suzhou Niumag Analytical Instrument Corporation in China. The strength range of the magnetic field is 0.3 ± 0.05 T, and the instrument’s main frequency is 12.8 MHz. The diameter of the probe coil is 60 mm. In the microscopic characteristic test, the central part of the specimen block after final destruction is scanned by electron microscopy. The microscopic morphological characteristics of the surface are obtained after 100 times magnification. The entire process of the test is shown in Figure 1.

## 3. Pore Radius Dividing and Fractal Dimension Calculating

### 3.1. Pore Radius Dividing

According to the principle of NMR [23], the surface relaxation of pore water inside porous materials can be expressed as:

(3) $$\frac{1}{T_{2}}=\rho_{2}\frac{S}{V}$$

In Equation (3), $T_{2}$ is the relaxation time, $\rho_{2}$ is the surface relaxation strength of the porous material, and its value is mainly determined by the mineral composition of the rock and the pore surface properties. S is the surface area of the pore, and V is the pore volume. During the NMR test, the pore shape is generally regarded as a spherical pore. Equation (3) can be reduced to:

(4) $$\frac{1}{T_{2}}=\rho_{2}\frac{F_{s}}{r_{c}}$$

In Equation (4), $F_{s}$ is the pore shape factor (spherical pore, $F_{s}$ = 3, cylindrical pore, $F_{s}$ = 2) and $r_{c}$ is the pore radius. $\rho_{2}$ and $F_{s}$ are constants. Because the pore shape is spherical, so the value of $F_{s}$ is 3. Equation (4) can be reduced to:

(5) $$r_{c}=CT_{2}$$

According to Equation (5), the pore radius ($r_{c}$) and the relaxation time ($T_{2}$) have a linear relationship and correspond to each other. The relaxation time distribution is also the pore radius distribution. The value of C will have a direct impact on the conversion of $T_{2}$ and $r_{c}$. Referring to the results of the current pore radius conversion study of cement mortar material [31,32], the value of C selected for the test is 0.01 μm/ms. Equation (5) can be reduced to:

(6) $$r_{c}=0.01T_{2}$$

**Figure 2:**
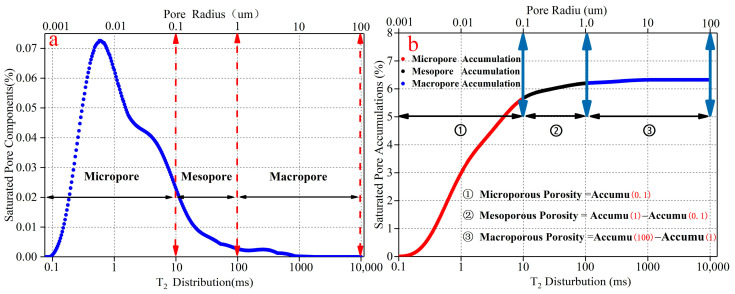
Pore radius dividing and porosity dividing. (**a**) is the pore radius dividing. (**b**) is the porosity dividing.

In the study of the division of pore radius in rocks, many experts and scholars have proposed various pore size division methods. In this paper, referring to Zhang’s [19] and Yan’s [33] methods for dividing the radius of rock pores, the microscopic pores inside rock-like material are divided into the following three types: micropore (pore radius < 0.1 μm), mesopore (0.1 μm < pore radius < 1 μm) and macropore (1 μm < pore radius < 100 μm). The division result is shown in Figure 2a.

Figure 2b shows the accumulation curve of saturated pores inside rock-like material detected by the NMR analysis system. The curve in the figure is the cumulative value of saturated pores. As the relaxation time ($T_{2}$) increases, the number of pores detected by NMR increases, and the cumulative value also increases. When the cumulative value reaches the maximum value, the cumulative value at this time is the porosity. According to the above pore size division, it can be seen that the relaxation time corresponds to the pore radius. The relaxation time changes so do the pore radius. If the relaxation time is converted to a pore radius, the pore accumulation values for different relaxation time intervals are also the pore accumulation values in different pore radius intervals. Based on the basis of the division of pore radius, the porosity can also be divided into three types with the same dividing way as the porosity of different radii: microporous porosity, mesoporous porosity, macroporous porosity.

### 3.2. Fractal Dimension Calculating

SEM is a method for detecting the surface micro-characteristic distribution of porous materials in two-dimensional space. However, its application in the field of micropore structure characterization is limited by the lack of quantification. Nevertheless, fractal features may exist in irregular surface microstructures magnified by electron microscope scanning. The complexity of surface micro-features can be quantified by calculating fractal dimensions. Among the many ways of representing the fractal dimension of electron microscope images, the box fractal dimension is widely used because of its simplicity, low difficulty and accurate calculation [12]. The evaluation expression is as follows:

(7) $$D_{s}=\underset{r\to 0}{\lim}\frac{\log N_{\left(r\right)}}{\log\left(1/r\right)}$$

In Equation (7), $D_{s}$ is the box fractal dimension. r is the edge length of the box that covers the entire image square. $N_{\left(r\right)}$ is the number of square boxes with r needed to cover the entire image. According to the calculation principle, when the side length of a square box is smaller, more boxes are required. When the side length of a square box is larger, fewer boxes are required.

**Figure 3:**
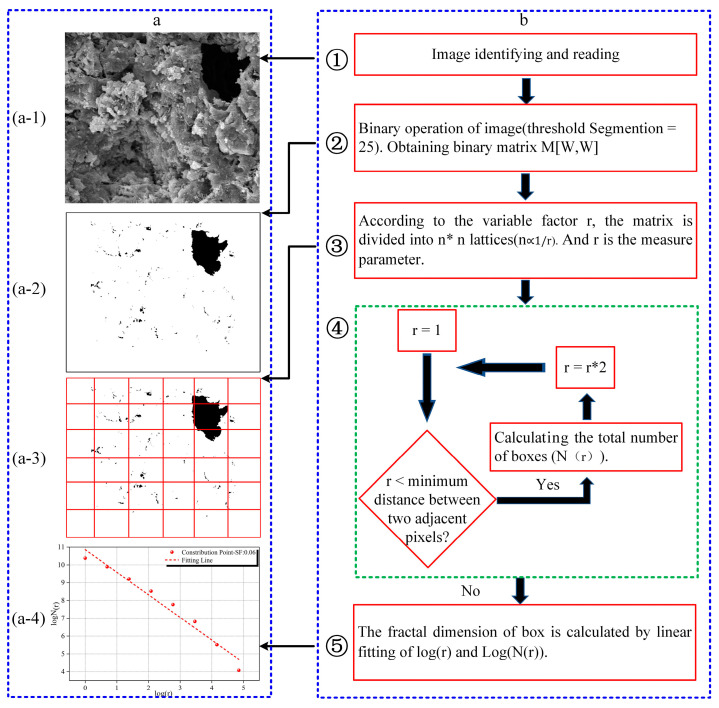
Box fractal dimension calculating. (**a**) is a schematic diagram of calculating fractal dimension. (**b**) is the calculation process of fractal dimension.

**Figure 4:**
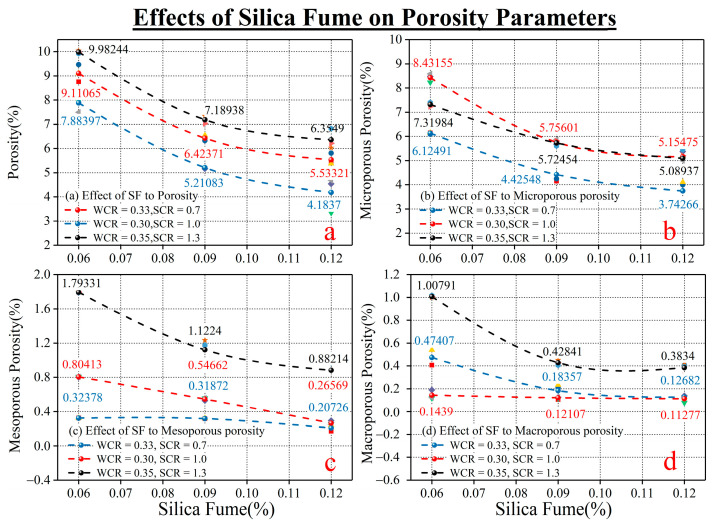
The influence of SF on porosities of different radii. (**a**) is the influence of SF on the porosity. (**b**) is the influence of SF on the microporosity. (**c**) is the influence of SF on the mesoporosity. (**d**) is the influence of SF on the microporosity.

Figure 3 is the calculation process for the box fractal dimension based on the scanning electron microscope image. As can be seen from the figure, the calculation program first recognizes and reads an image (Figure 3(a-1)). Next, the binary image is calculated, and the previously identified image is transformed into a matrix M with only 0 and 1 (Figure 3(a-2)). The value of the binarization threshold for this image is 25 in this test. After binary calculations, the solids and pores in the internal microstructure are given 0 and 1, respectively. It can be seen from Figure 3(a-2) that the image can completely distinguish between pores and solids inside the material. Then, the image (Figure 3(a-3)) matrix is divided into n∗n (n∝1/r) squares by the square box side length (r). The starting value of r is 1, and then r increases in the range of r=r∗2 until r reaches the minimum distance between two adjacent pixels to stop. In each calculation, the value of the number of square boxes $\left(N_{\left(r\right)}\right)$ only depends on the calculation result of the box covering the assignment 0 area in the operation program. Finally, 1/r and $N_{\left(r\right)}$ obtained by the calculation of each value of r are performed logarithmic operations. The logarithmic coordinates are linearly fitted, and the absolute value of the slope of the fitted line is the box fractal dimension (Figure 3(a-4)).

## 4. Results and Discussions

### 4.1. Effect of SF on Micro-Pore Structure

Figure 4 is the variation law of SF on the porosity of different radii of rock-like material under different conditions of WCR and SCR. As can be seen from Figure 4a, despite being under different conditions, the porosity shows a decreasing trend with the increase in SF. As depicted in Figure 4b–d, except for the mesoporous porosity and macroporous porosity under the condition of WCR = 0.33 and SCR = 0.7, the microporous structure is dominated by micropore and followed by mesopore. Micropore is the least. In the variation in porosity parameters of different radii, the porosity with different conditions of WCR and SCR decreased gradually when the amount of SF was added. However, there are differences in the rate of reduction in porosity in different radii. Table 5 is the calculation statistics of the reduction rate of porosity of different radii. As can be seen from the table, the porosity of macroporous pores has the largest reduction rate, and the reduction rate reaches 52.18%. The reduction rate of mesoporous porosity is 51.25%, which is slightly lower than that of macroporous porosity. The reduction rate of microporous porosity (36.01%) is the smallest. The analysis shows that SF had a significant impact on the macropore and mesopore but was relatively low on the micropore.

Experimental phenomena showed that SF has obvious effects on the porosity of different radii. The SF used in this test had a particle size of 1 μm and a specific surface area of 80 m^2^/g, while the specific surface area of ordinary portland cement is 0.38 m^2^/g [9]. Thus, the specific surface area of SF is much larger than the specific surface area of the cement. The particle size of the SF is also much smaller than the particle size of the cement. When the amount of SF increased from 6% to 12%, the SF with smaller particle size entered the gap between the cement with larger particle size, filling the pores in the portland cement hydration product and reducing the porosity. Therefore, the porosity of different radii decreased with the increase in SF [34].

In addition, from Table 3, it can be seen that SF used in this test contains a large number of SiO_2_, whose content reaches 99.1%. SiO_2_ has volcanic ash activity and can react with calcium hydroxide in the hydration reaction product of portland cement. The chemical reaction equation is as follows [10,35,36]:

(8) $$2C_{3}S+6H\to C_{3}S_{2}H_{3}+3\mathrm{CH}$$

(9) $$2C_{2}S+4H\to C_{3}S_{2}H_{3}+\mathrm{CH}$$

(10) $$\mathrm{xCH}+{\mathrm{SiO}}_{2}+H_{2}O\to \mathrm{xCaO}\cdot {\mathrm{SiO}}_{2}\cdot {\mathrm{nH}}_{2}O$$

According to the above chemical reaction equation, portland cement is hydrated to form calcium silicate and calcium hydroxide. However, the calcium hydroxide generated by the hydration reaction and the silicon powder reacted with volcanic ash, which once again formed calcium silicate hydrate, increasing the content of calcium silicate in the interior of rock-like material [10,11]. These increased calcium silicate hydrations were connected and combined with the calcium silicate hydrate generated by the original cement hydration reaction to fill the internal voids. The formation of microscopic pores was decreased, and the porosity of rock-like material was also reduced

However, the main reason for the variation in porosity reduction in different radii may be the radius of porosity. Macropores have large pore radii, so SF and hydrated calcium silicate formed by volcanic ash reaction can easily enter the pores for filling. Due to the small pore radius, it is more difficult for SF and calcium silicate hydrate to enter the pore. Therefore the decrease in the porosity is smaller than the decrease in the porosity of macroporous porosity [37]. The radius of the micropore is the minimum of three different types of the pore, and the difficulty of entering the pore interior is the highest. Therefore the decrease in pore porosity is much smaller than that of medium and large porosity.

### 4.2. Effect of SF on Fractal Dimensions

**Figure 5:**
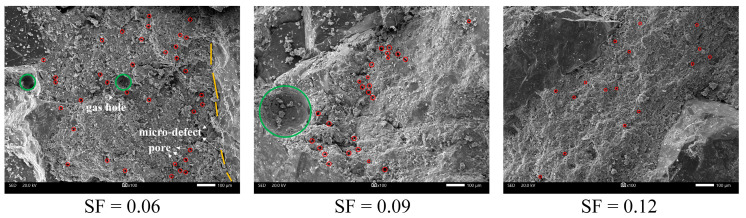
The internal microscopic characteristics of different SF magnified 100 times.

Figure 5 shows the internal microscopic characteristics of rock-like material under different SF conditions after being magnified 100 times by electron microscopy. It can be seen that SF has a significant influence on microscopic morphology. When the incorporated SF in this test is 6%, there are gas holes, pores and microcracks between quartz sand and hydration products on the internal surface. The number of pores is large, and the morphological distribution is disordered and irregular. When the SF is increased from 6% to 9%, the number of gas holes and pores decreases significantly. The pore morphological distribution gradually accumulates. In addition, the number of microcracks gradually disappears. When the SF is increased from 9% to 12%, the gas holes and micro-cracks basically disappear, and the number of pores is also greatly reduced. The internal surface is flat, dense and evenly distributed. The characteristic transformation of the internal surface from porous, rough to dense, flat and uniform also confirmed the influence of SF on porosity. With the continuous increase in SF, the number of microscopic pores gradually decreased, and the porosity decreased. The internal surface distribution was flat and uniform.

**Figure 6:**
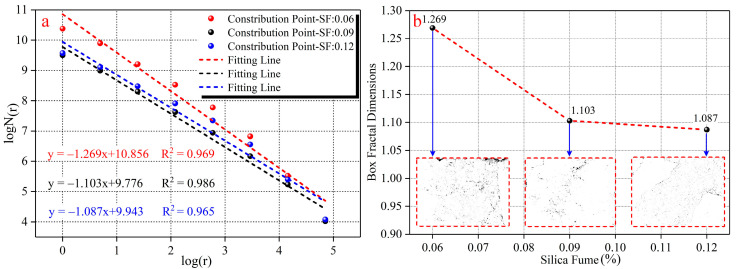
The influence of SF on fractal dimensions. (**a**) is the fitting calculation results of fractal dimension. (**b**) is the influence of SF on fractal dimension.

Fractal dimension is a robust value that can be used to characterize the microporous structure of cementitious materials under different experimental ratio conditions (WCR, SCR, ADR) [38,39,40], which can quantify the complexity of the microporous structure under certain scale conditions. Figure 6a shows the fitting calculation result of fractal dimensions under different SF conditions. According to the calculation results, the value of the fractal dimension is above 1.10. Under each experimental condition, $\log_{\left(r\right)}$ and $\log\;N_{\left(r\right)}$ conform to a good linear relationship, whose correlation coefficient is above 0.96. The data show that the spatial distribution of microscopic pores in rock-like material under different SF conditions has fractal characteristics within a certain range. Figure 6b shows the influence of SF on fractal dimensions. It can be seen from the binary image in the figure when the amount of SF is increased from 6% to 12%, the microscopic characteristics of the internal surface of the material change from the number of pores and irregular morphological distribution to the small number of pores and the regularity of morphological distribution. At the same time, the fractal dimension shows a gradually decreasing trend with the increase in SF. According to fractal theory [41], the box fractal dimension can characterize the irregularity of microscopic features of the internal surface of porous materials at a certain measurement scale. The larger the fractal dimension is, the more irregular the micropore distribution on the inner surface becomes. The smaller the fractal dimension is, the more regular the micropore distribution is on the inner surface. Based on the effect of SF on porosity and SEM micro-characteristics, when the amount of SF was the minimum (6%), the porosity was the largest, and the pore morphology was the most complex, so the fractal dimension was the largest. When the amount of SF was added, the porosity gradually decreased. The morphological distribution of microscopic pores on the surface gradually changed from complex disorder to uniform rules. Therefore, the law of gradually decreasing box fractal dimensions occurs.

### 4.3. Correlation Analysis between Mechanical Properties and Porosity and Fractal Dimension

**Figure 7:**
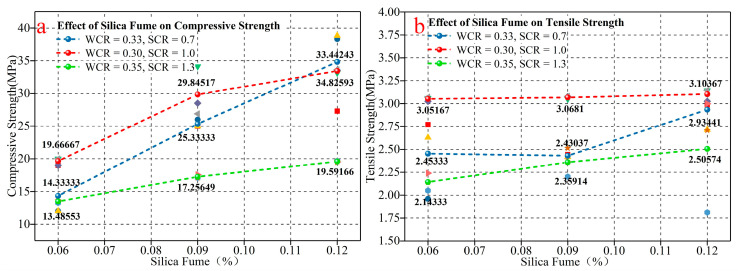
The influence of SF on mechanical properties. (**a**) is the influence of SF on the compressive strength. (**b**) is the influence of SF on the tensile strength.

Figure 7 shows the influence of SF on the mechanical properties of the rock-like material. When the amount of SF incorporated into the test gradually increases, the uniaxial compressive strength and tensile strength under different WCR and SCR conditions show a gradually increasing trend. Table 6 shows increase rates of compressive strength and tensile strength under different conditions of SF. Among them, the average increase rate of compressive strength is 85.22%, and the average increasing rate of tensile strength is 12.74%. The data prove there is a significant enhancement effect of SF on mechanical properties. In accordance with the influence of SF on the microporous structure, the SF physically filled the pores between cement particles. Volcanic ash reaction also occurred to form calcium silicate hydrate, which combined the original hydration products and filled the pores, improving the internal compactness and reducing the porosity. Thus, the compressive strength and tensile strength are enhanced.

**Figure 8:**
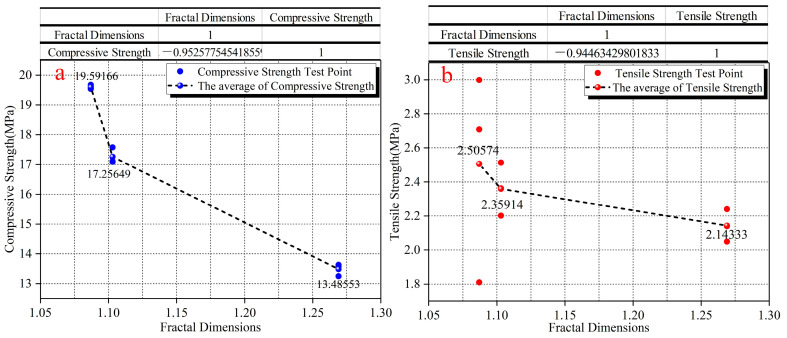
The influence of fractal dimension on compressive strength and tensile strength. (**a**) is the influence of fractal dimension on compressive strength. (**b**) is the influence.

**Figure 9:**
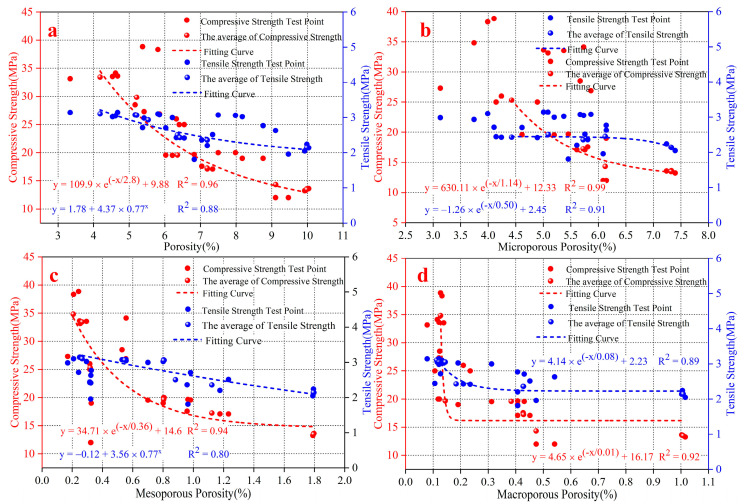
Fitting analysis of different porosity parameters and mechanical properties. (**a**) is the fitting analysis of porosity and mechanical properties. (**b**) is the fitting analysis of microporosity and mechanical properties. (**c**) is the fitting analysis of mesoporosity and mechanical properties. (**d**) is the fitting analysis of macroporosity and mechanical properties.

Based on all the above research contents, it can be seen that SF had a corresponding influence on the microscopic pore structure and macroscopic mechanical properties. Therefore, to verify whether there is an intrinsic correlation between microscopic porosity parameters and macroscopic mechanical properties, the correlation coefficient calculation and fitting analysis between mechanical properties and the microscopic porosity parameters and fractal dimensions were carried out. The calculation results are shown in Table 7, Figure 8 and Figure 9. The correlation coefficient (r) is a quantification of the correlation between two variables in correlation analysis with a calculated values range of [–1,1]. When the value of r is positive, the correlation is positively correlated. The values of the two variables increase together. When the value of r is negative, the correlation is negative. As one variable increases, the other decreases. In this analysis, the paper divides correlation coefficient into four levels: the weak correlation (0.2 ≤ |r| ≤ 0.4), the moderate correlation (0.4 ≤ |r| ≤ 0.6), the strong correlation (0.6 ≤ |r| ≤ 0.8) and the extremely strong correlation (0.8 ≤ |r| ≤ 1.0). Figure 8 shows the influence law of fractal dimensions on the mechanical properties. There is a strong negative correlation between fractal dimension and compressive strength, and tensile strength. The correlation coefficients are above 0.94. With the continuous increase in fractal dimensions, the compressive strength and tensile strength show a gradual decrease in variation.

Table 7 shows the calculation results of the correlation between different porosity parameters and mechanical properties of a rock-like material. The results show the correlation between different porosities and compressive strength, and tensile strength is negatively correlated, but the correlation coefficients are different. The porosity, microporous porosity and mesoporous porosity are extremely strong and negatively correlated with mechanical strengths, but the correlation coefficients between different porosity parameters and compressive strength (r > 0.90) are higher than their correlation coefficients with tensile strength (r < 0.89). However, the correlation between macroporous porosity and mechanical property is negatively correlated strongly. The correlation coefficient with tensile strength is greater than the correlation coefficient with compressive strength, which is contrary to the laws of other porosity parameters and mechanical properties. Figure 9 shows the fitting calculation of different porosity parameters and mechanical properties. The compressive strength and tensile strength have a good exponential relationship with different porosity parameters. Mechanical properties show exponentially decreasing laws with the increase in different porosity parameters. However, the fitting correlation coefficients of different porosity parameters and compressive strength (R^2 > 0.92) are higher than the correlation coefficients with tensile strength (R^2 < 0.91). Based on the correlation coefficient and the fitting relationship, the macroporous porosity and compressive strength with the smallest correlation coefficient also had the smallest correlation coefficient in the fitting relationship. However, the tensile strength did not seem to accord with this law. The microporous porosity with the least correlation coefficient did not have the least fitting correlation coefficient.

The microscopic pore structure parameters of rock-like material performed an extremely important role in the influence of SF on mechanical properties. The change in SF directly affected the change in the morphology and quantity of microscopic pore distribution. When the incorporation of SF was small, the number of pores inside the material was large. The surface was rough, and the morphological distribution was complex and disorderly. The box fractal dimension was at the maximum. When the specimen was subjected to force, the pores in the specimen deformed and expanded, and the different pores were connected and penetrated. The rupture was accelerated, which eventually led to a decrease in the mechanical properties. When the amount of SF was added, the internal pores were filled. The number of pores was reduced, and the porosity was reduced. The surface distribution was flat, uniform and dense. Meanwhile, the box fractal dimension was at its minimum. This evenly distributed and dense structure enhanced the mechanical properties of the material.

## 5. Conclusions

Based on the combination of NMR and SEM, in this paper, the effects of micron-SF on microscopic pore structure and macroscopic mechanical properties were analyzed under different conditions of WCR and SCR. The intrinsic relationship between microporous pore structure and macroscopic mechanical properties was discussed. The main conclusions are as follows:

(1) Although under different conditions of WCR and SCR, the porosity of different radii showed a decreasing trend with the increase in SF. Among the porosity reduction rates, the macroporous porosity reduction rate was the largest, and the microporous porosity was the smallest;
(2) When the incorporation of SF increased from 6% to 12%, the microscopic characteristics changed from a large number of pores, complex and disorderly morphological distribution, rough surface to a small number of pores, regular morphological distribution, flat surface, uniformity and denseness. The box fractal dimension also showed a gradually decreasing change law;
(3) Microscopic pore structure played an important role in the influence of SF on mechanical properties. The compressive strength and tensile strength showed an increasing trend with the increase in SF. There was a negative correlation between the mechanical characteristic parameters and the fractal dimension, and the porosity parameters of different radii. Moreover, the compressive strength and tensile strength were in a good exponential relationship with porosity parameters of different radii.

## Figures and Tables

**Table 1:** Chemical composition of portland cement.

Chemical Composition	3CaO·SiO_2_	2CaO·SiO_2_	3CaO·Al_2_O_3_	4CaO·Al_2_O_3_·Fe_2_O_3_
Content	52.8%	20.7%	11.5%	8.8%

**Table 2:** Detailed parameters of rock-like material.

Material	Traits	Main Ingredients	Particle Size	Density (g/cm^3^)
Quartz sand	Yellow and white particles	quartz > 99%	0.5–1.0 mm	1.49
Naphthalene water reducer	Brown yellow powder	β-Naphthal-enesulfonate sodium formaldehyde condensate	-	-

**Table 3:** The component content of silica fume.

Material	Traits	Particle Size	Density (g/cm^3^)	Specific Surface Area (m^2^/g)	Component Content						
					CaO	SiO_2_	Al_2_O_3_	CuO	MgO	Na_2_O	K_2_O
Silica Fume	White powder	1 μm	2.2–2.6	80	0.1	99.2	0.2	0.1	0.2	0.1	0.2

**Table 4:** Exerimental Scheme.

Experimental Schemes	Experimental Factors		
	SF(SF/WCR)	WCR	SCR
1	6%	0.3	1
	9%		
	12%		
2	6%	0.33	0.7
	9%		
	12%		
3	6%	0.35	1.3
	9%		
	12%		

**Table 5:** The reduction rate of different types of porosity.

Porosity Type	Silica Fume			Reduction Rate	The Average of Reduction Rate
	6%	9%	12%		
Microporous porosity	6.12491	4.42548	3.74266	38.89%	36.01%
	8.43155	5.75601	5.15475	38.86%	
	7.31984	5.72454	5.08937	30.47%	
Mesoporous porosity	0.32378	0.31872	0.20726	35.99%	51.25%
	0.80413	0.54662	0.26569	66.96%	
	1.79331	1.1224	0.88214	50.81%	
Macroporous porosity	0.47407	0.18357	0.12682	73.25%	52.28%
	0.1439	0.12107	0.11277	21.63%	
	1.00791	0.42841	0.3834	61.96%	

**Table 6:** The increase rate of different mechanical properties.

Mechanical Property	Silica Fume			Increase Rate	The Average of Increase Rate
	6%	9%	12%		
Compressive strength (MPa)	19.6667	29.84517	34.82593	77.08%	85.22%
	14.33333	25.3333	33.44243	133.32%	
	13.48553	17.25649	19.59166	45.28%	
Tensile strength (MPa)	3.05167	3.0681	3.10367	1.70%	12.74%
	2.45333	2.43037	2.93441	19.61%	
	2.14333	2.35914	2.50574	16.91%	

**Table 7:** Correlation coefficients between different porosity and mechanical properties.

Parameter Type	Silica Fume	
	Compressive Strength (MPa)	Tensile Strength (MPa)
Porosity	−0.9339	−0.8962
Microporous Porosity	−0.9242	−0.8621
Mesoporous Porosity	−0.9064	−0.8937
Macroporous Porosity	−0.6778	−0.7985

## Data Availability

The data presented in this study are available on request from the corresponding author.

## Symbols and Notations

WCR	Water–cement ratio
SCR	Sand–cement ratio
ADR	Admixtures
SF	Silica Fume
NMR	Nuclear Magnetic Resonance
SEM	Scanning Electron Microscope
MIP	Mercury Injection Experiment
CT	Computed Tomography
$C_{3}S$	3CaO·SiO_2_
$C_{2}S$	2CaO·SiO_2_
CH	Ca(OH)_2_
H	H_2_O
$C_{3}S_{2}H_{3}$	3CaO·SiO_2_·3H_2_O
